# Rescue subthalamic stimulation after unsatisfactory outcome of pallidal stimulation in Parkinson's disease: a case series and review

**DOI:** 10.3389/fnagi.2023.1323541

**Published:** 2024-01-09

**Authors:** Zhitong Zeng, Peng Huang, Zhengyu Lin, Yixin Pan, Xiaonan Wan, Chencheng Zhang, Bomin Sun, Dianyou Li

**Affiliations:** Department of Neurosurgery, Center for Functional Neurosurgery, Ruijin Hospital, Shanghai Jiao Tong University School of Medicine, Shanghai, China

**Keywords:** Parkinson's disease, deep brain stimulation, subthalamic nucleus, globus pallidus interna, rescue therapy

## Abstract

**Background:**

Subthalamic nucleus (STN) and globus pallidus interna (GPi) are two main structures primarily targeted by deep brain stimulation (DBS) to treat advanced Parkinson's disease (PD). A subset of cases with unsatisfactory outcomes may benefit from rescue DBS surgery targeting another structure, while these patients' characteristics have not been well described and this phenomenon has not been well reviewed.

**Methods:**

This monocentric retrospective study included patients with PD, who underwent rescue STN DBS following an unsatisfactory outcome of the initial bilateral GPi DBS in a retrospective manner. A short review of the current literature was conducted to report the clinical outcome of rescue DBS surgeries.

**Results:**

Eight patients were identified, and six of them were included in this study. The rescue STN DBS was performed 19.8 months after the initial GPi DBS. After 8.8 months from the rescue STN DBS, patients showed a significant off-medication improvement by 29.2% in motor symptoms compared to initial GPi DBS. Non-motor symptoms and the health-related quality of life were also significantly improved.

**Conclusion:**

Our findings suggest that the rescue STN DBS may improve off-medication motor and non-motor symptoms and quality of life in patients with failure of initial GPi DBS. The short review of the current literature showed that the target switching from GPi to STN was mainly due to poor initial outcomes and was performed by target substitution, whereas the switching from STN to GPi was mainly due to a gradual waning of benefits, long-term axial symptoms, dyskinesia, and dystonia and was performed by target addition.

## Introduction

The subthalamic nucleus (STN) and the globus pallidus interna (GPi) are two well-established deep brain stimulation (DBS) targets in the treatment of advanced Parkinson's disease (PD). Consistent and comparable overall motor benefits of both targets observed in multiple randomized controlled studies suggest that identifying a definitive optimal target between the two remains elusive (Follett et al., 2010; Odekerken, 2013; Ramirez-Zamora and Ostrem, 2018; Sobesky, 2022). The STN and GPi are closely interconnected anatomically, and both serve as crucial integrator hubs in indirect and hyperdirect pathways. Consequently, a hypothesis suggesting that both STN DBS and GPi DBS might modulate an “overlapping” functional network has been proposed and preliminarily validated (Sobesky, 2022). This hypothesis could be one of the reasons for the observed similarities in the overall therapeutic effects of STN DBS and GPi DBS. However, this does not imply that modulating either target would lead to identical effects (Sobesky, 2022); indeed, subtle differences in various symptoms have been well-documented in the literature. GPi DBS may have potential advantages compared to STN DBS in improving dyskinesia (Odekerken, 2013; Zhang, 2021), gait impairment, and axial symptoms (Obeso et al., 2001; St George et al., 2012; Vercruysse et al., 2014). In contrast, STN DBS may be preferred over GPi DBS in reducing anti-parkinsonian medication and related complications (Krack et al., 2003; Moro, 2010; Ramirez-Zamora and Ostrem, 2018). In addition, STN stimulation may be more effective in the motor symptoms during the off-medication phase, whereas GPi stimulation might show greater efficacy during the on-medication phase (St George et al., 2010). These efficacy differences make tailored target selection both a necessity and a challenge.

Several studies have reported that a subset of PD patients experienced an initial lack or waning of DBS efficacy despite satisfactory DBS lead placement and optimal adjustment of medication and stimulation parameters (Allert et al., 1996, 2012; Houeto et al., 2000; Volkmann et al., 2004; Minafra et al., 2014; Cook, 2015; Matias et al., 2016; Brinke et al., 2018; Zhang et al., 2019). In these cases, the rescue surgery with reoperation to another target, either switching from GPi to STN (Allert et al., 1996; Houeto et al., 2000; Volkmann et al., 2004; Brinke et al., 2018) or vice versa (Allert et al., 2012; Minafra et al., 2014; Cook, 2015; Matias et al., 2016; Zhang et al., 2019), might provide benefits. Two types of reoperation procedures have been previously reported, i.e., adding leads to another target (Volkmann et al., 2004; Allert et al., 2012; Minafra et al., 2014; Cook, 2015; Matias et al., 2016) and changing the target to another (Allert et al., 1996; Houeto et al., 2000; Brinke et al., 2018; Zhang et al., 2019). However, the suitability of patients for reoperation and the criteria for choosing the type of reoperation remain unclear.

In this study, we reported the clinical outcome of PD patients, who underwent rescue STN DBS following an unsatisfactory outcome of the initial GPi DBS in a retrospective manner. The rescue STN DBS could improve off-medication motor and non-motor symptoms and the health-related quality of life, with a more significant reduction in the anti-parkinsonian medication. In addition, a review of rescue DBS by target switching or target addition was provided. We concluded that the rescue STN for GPi DBS was primarily due to suboptimal initial efficacy and was performed through target switching. Conversely, the rescue GPi for STN DBS was predominantly due to a gradual decline in benefits, long-term axial symptoms, dyskinesia, and dystonia and was performed through target addition.

## Methods

### Participants

We reviewed our PD clinical database with a timeframe ranging from July 2017 to March 2020 and retrieved data from patients who underwent the first DBS surgery but required reoperation to another target in this period. A total of 170 GPi DBS surgeries were performed during the above-mentioned period. Eight patients underwent reoperation to STN due to unsatisfactory efficacy of initial GPi DBS. These patients had initially received GPi DBS because both STN and GPi were considered to have similar benefits in overall motor symptoms during that period, with GPi improving axial symptoms more advantageously (Ramirez-Zamora and Ostrem, 2018). Increased battery consumption was one of the disadvantages of the GPi DBS. However, around that time, rechargeable batteries became available in China, addressing this issue.

The inclusion criteria for PD DBS surgeries performed in our center were as follows (Lin, 2021): (1) clinical diagnosis of idiopathic PD according to the MDS Clinical Diagnostic Criteria (Postuma et al., 2015); (2) the presence of disabling motor fluctuations, wearing-off phenomena, and/or dyskinesia; (3) approximately 24% improvement in the MDS Unified Parkinson's Disease Rating Scale part III (MDS UPDRS-III) total score following a supra-threshold levodopa challenge test (LCT) (Merello et al., 2011); and (4) the absence of comorbidities, including dementia or severe neuropsychiatric disorders. All patients included in this study had discontinued GPi DBS, and they were reoperated for STN due to unsatisfactory efficacy of motor symptoms despite optimal medication and stimulation parameter adjustments. The unsatisfactory efficacy of initial GPi DBS was defined by the following criteria: (1) the off-medication improvement rate, which is lower than 30% of the preoperative levodopa responsiveness measured by MDS UDPRS-III; (2) the persistence of motor complications, which did not improve after parameter adjustments; and (3) patient-reported dissatisfaction with main initial complaints remaining unresolved after GPi DBS. Six of the eight patients were participated in this study. One patient was excluded due to concurrent cingulotomy performance, and another patient refused to participate.

### Surgical procedure

The surgical procedure of the rescue DBS surgery has been published elsewhere (Zhang et al., 2019). Specifically, for the rescue DBS surgery, electrodes were bilaterally implanted into the STN (3387; Medtronic, Minneapolis, MN) through the same burr holes after the removal of GPi leads (3387; Medtronic, Minneapolis, MN). Leads were reconstructed in the Montreal Neurological Institute (MNI) space using a standardized pipeline implemented in the LeadDBS toolbox (version 2.6, MATLAB 2021b) (Supplementary Figure 1).

### Clinical outcome assessment

Motor functions were clinically assessed at the baseline in both off- and on-medication conditions, as well as before and after STN DBS surgery (i.e., post-GPi and post-STN, respectively) in four conditions (off-stimulation, on-stimulation, off-medication, and on-medication). Before the assessment, anti-parkinsonism medication was withdrawn for 12 h (usually overnight), and bilateral stimulation was turned off and washed out for ~1 h. The motor symptoms were first assessed in the off-stimulation/off-medication condition. Afterward, the stimulation was turned on for 1 h, and the assessment was performed in the on-stimulation and off-medication conditions. For the on-medication condition, a single dose of levodopa was administered twice subsequently to reach the state in which both patient and examiner agreed that the best functional state was achieved; then, motor assessments were successively performed in off-stimulation and on-stimulation conditions. Of note, a supra-threshold dose (the usual effective dose taken in the morning × 1.5) was administrated for the baseline and post-GPi evaluation, respectively, and a standard first-morning levodopa dose was administrated for post-STN evaluation. Motor assessments were videotaped and blinded to the rater. The primary outcome was the change in the MDS UPDRS-III total score between the baseline and post-STN evaluation visit (EV). A series of Parkinsonian symptoms were individually analyzed: (1) rigidity (item 3.3); (2) bradykinesia (item 3.2, 3.4–3.8, 3.14); (3) tremor (item 3.15–3.18); and (4) axial signs (item 3.9–3.13).

Second outcomes included the global cognitive function measured by the Montreal Cognitive Assessment (MoCA) and the Mini-Mental State Examination (MMSE), non-motor symptoms measured by the Non-Motor Symptoms Scale (NMSS), PD-related voice impairment measured by the 10-item Voice Handicap Index (VHI-10), gait and falls risk measured by the Gait and Falls Questionnaire (GFQ), and the health-related quality of life measured by the short-form 8-item Parkinson's Disease Questionnaire (PDQ-8). Given that our center combined MMSE and MoCA for cognitive function assessment at this stage, we converted the MMSE score to MoCA for comparability (Melikyan et al., 2021). Specifically, the three MMSE scores 26, 28, and 29 were equated to MoCA scores 21, 25, and 26, respectively. The levodopa equivalent daily dose (LEDD) was also calculated.

### Statistical analysis

The Friedman test was used for comparisons between three time points (i.e., baseline, post-GPi, and post-STN), with multiple comparisons corrected by the False Discovery Rate control. For comparisons between off-stimulation and on-stimulation conditions at post-GPi and post-STN EVs, the two-tailed paired-sample *t*-test or Wilcoxon Signed-rank test was applied based on the normality of data tested by the Shapiro–Wilk test. A *p*-value of < 0.05 was considered statistically significant. Adapted from Brinke et al. (2018), patients were considered as responders if there was an off-medication/on-STN improvement of ≥ 24% (Merello et al., 2011) in the MDS UPDRS-III score after reoperation compared with the off-medication/on-GPi condition. Statistical analyses were conducted using R-version 4.0.2.

## Results

### Demographic characteristics

The demographic characteristics of this cohort are shown in Table 1. The mean age was 52.5 ± 11.2 (range: 36–63) years, and the mean disease duration was 9.8 ± 2.1 (range: 8–13) years at the time of the initial GPi DBS surgery. All patients received STN DBS for a mean time interval of 19.8 ± 8.0 (range 10–32) months after initial GPi DBS surgery. These patients opted for the rescue surgery mainly due to the unsatisfactory efficacy of initial GPi DBS on rigidity and bradykinesia (Supplementary Table 1).

**Table 1:** Demographic and clinical characteristics.

**Characteristics**	**Value (*N* = 6)**
Sex, M (%)	5 (83.3)
Age (year)	52.5 ± 11.2
Disease duration (year)	9.8 ± 2.1
Off-medication MDS UPDRS III Score at baseline	58.1 ± 17.6
LCT response at baseline	59.2% ± 11.7%
LCT response before reoperation	53.7% ± 7.5%
Time between initial GPi DBS and rescue STN DBS (month)	19.8 ± 8.0
Follow-up after rescue STN DBS (month)	8.83 ± 3.37

Values were presented as mean ± standard deviation.

LCT, levodopa challenge test; GPi, globus pallidus interna; STN, subthalamic nucleus; DBS, deep brain stimulation.

### Target accuracy, stimulation parameters, and medication

The electrode locations for STN and GPi were reconstructed and visualized in MNI space (Supplementary Figure 1). We optimized GPi DBS parameters prior to the rescue surgery. At each follow-up visit, the integrity of the DBS system was verified to exclude the hardware-related abnormality. Supplementary Table 2 shows the final DBS parameters used for initial GPi stimulation and for STN stimulation, respectively. We used high-frequency, monopolar, or double monopolar stimulation with pulse widths between 60 and 90 μs. These parameter settings were similar to those reported in previous GPi or STN DBS clinical trials (Ramirez-Zamora and Ostrem, 2018).

### Primary outcome

**Figure 1:**
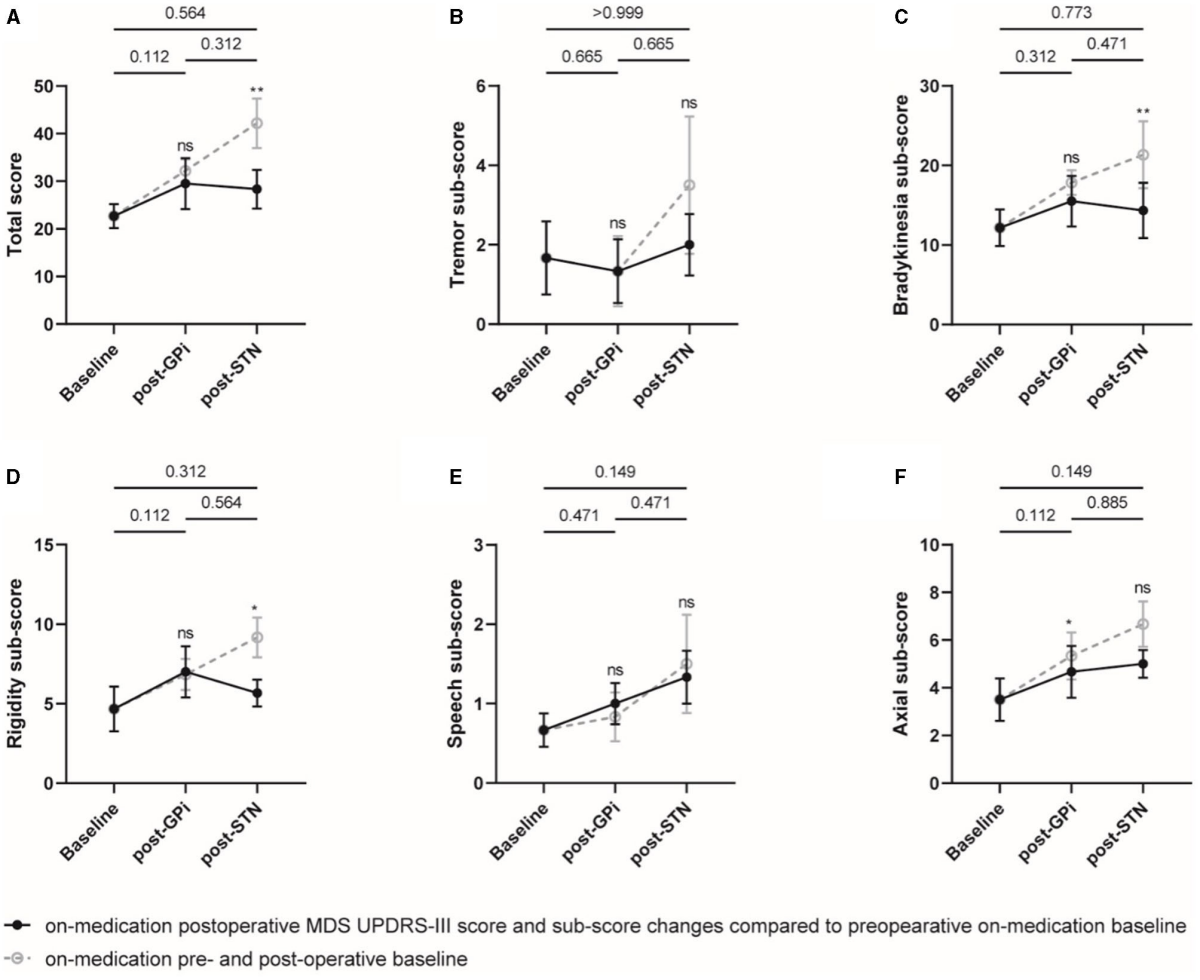
Off-medication MDS UPDRS-III total score and sub-scores at different evaluation visits. The total score **(A)** and sub-scores of the tremor **(B)**, bradykinesia **(C)**, rigidity **(D)**, speech **(E)**, and axial symptoms **(F)** were plotted, respectively. Individual data are represented as black dots or circles, with error bars for the standard error. The *p*-value and ns on the horizontal line represented the statistical significance between baseline, post-GPi and post-STN (on-stimulation), while the ones on the post-GPi and post-STN time points represented the statistical significance between on- and off-stimulation conditions. *p-value < 0.05, **p-value < 0.01.

The initial GPi DBS did not significantly improve the off-medication MDS UPDRS-III total score compared to the baseline. However, the rescue STN DBS demonstrated a significant improvement with a mean of 37.5% (*p* = 0.002) and 29.2% (*p* = 0.030) compared to the baseline and initial GPi DBS, respectively. Four of the six patients were classified as responders with an improvement of ≥24% in the MDS UPDRS-III total score compared to GPi DBS. In terms of the MDS UPDRS-III sub-scores, reductions in bradykinesia, rigidity, and axial sign sub-scores were observed after rescue STN DBS compared to both baseline (improved by 31.5%, 48.1%, and 40.0%; *p* = 0.009, 0.021, and 0.061) and initial GPi DBS (improved by 27.5%, 42.0%, and 24.6%; *p* = 0.083, 0.043, and 0.248), respectively (Figure 1, Supplementary Table 3).

**Figure 2:**
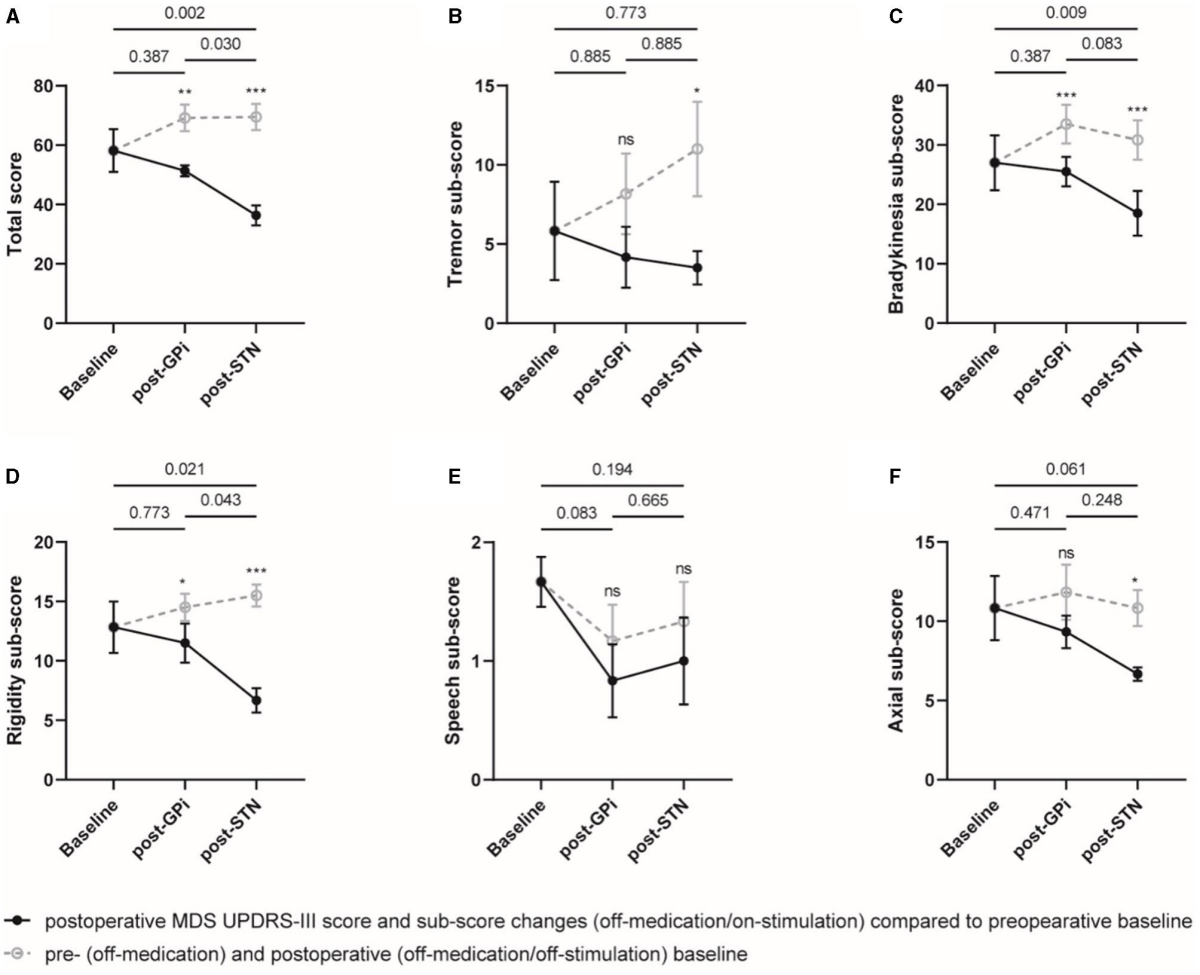
On-medication MDS UPDRS-III total score and sub-scores at different evaluation visits. The total score **(A)** and sub-scores of the tremor **(B)**, bradykinesia **(C)**, rigidity **(D)**, speech **(E)**, and axial symptoms **(F)** were plotted, respectively. Individual data are represented as black dots or circles, with error bars for the standard error. The *p*-value and ns on the horizontal line represented the statistical significance between baseline, post-GPi and post-STN (on-stimulation), while the ones on the post-GPi and post-STN time points represented the statistical significance between on- and off-stimulation conditions. *p-value < 0.05, **p-value < 0.01, ***p-value < 0.001.

On the other hand, compared to the initial GPi DBS, the rescue STN DBS did not significantly improve the MDS UPDRS-III total score and sub-scores in the on-medication condition, probably due to a dose reduction in antiparkinsonian medications. However, significant improvements in total score and sub-scores of bradykinesia and rigidity were observed with STN DBS compared to the postoperative off-stimulation and on-medication conditions (Figure 2, Supplementary Table 4).

### Secondary outcomes

Compared to the baseline (79.2 ± 30.1), the NMSS score showed a statistically significant improvement with rescue STN DBS (46.7 ± 33.1, *p* = 0.009), whereas there was no significant improvement with initial GPi DBS (91.8 ± 29.0, *p* = 0.387). Similarly, the GFQ score was significantly ameliorated by 48.0% (post-STN: 10.5 ± 8.6 vs. baseline: 21.2 ± 13.6, *p* = 0.014) after rescue STN DBS compared to the baseline. In addition, the rescue STN DBS significantly reduced the PDQ-8 score by 45.3% (post-STN: 9.3 ± 4.2 vs. baseline: 17.0 ± 3.8, *p* = 0.006) and the LEDD by 37.2% (post-STN: 525.0 ± 117.3 vs. baseline: 836.5 ± 158.4, *p* = 0.03) compared to the initial GPi DBS (Table 2).

**Table 2:** Second outcomes at different evaluation visits.

**Outcomes**	**Baseline**	**GPi DBS**	**ΔGPi-baseline**	***p-*value**	**STN DBS**	**ΔSTN-baseline**	***P-*value**	**ΔSTN-GPi**	***p-*value**
MoCA	22.0 ± 1.5	22.7 ± 2.3	3.2%	0.564	25.3 ± 2.5	15.0%	0.471	11.5%	0.885
NMSS	79.2 ± 30.1	91.8 ± 29.0	15.9%	0.387	46.7 ± 33.1	−41.0%	0.083	−49.1%	0.009^*^
GFQ	21.2 ± 13.6	20.2 ± 16.8	−4.7%	0.312	10.5 ± 8.6	−50.5%	0.014^*^	−48.0%	0.149
VHI	8.0 ± 16.0	16.3 ± 14.7	103.8%	0.312	19.3 ± 11.8	141.3%	0.248	18.4%	0.885
PDQ-8	15.3 ± 4.0	17.0 ± 3.8	11.1%	0.248	9.3 ± 4.2	−39.2%	0.112	−45.3%	0.006^*^
LEDD	777.4 ± 290.3	836.5 ± 158.4	7.6%	0.665	525.0 ± 117.3	−32.5%	0.083	−37.2%	0.030^*^

Values were presented as mean ± standard deviation.

MoCA, Montreal Cognitive Assessment; MMSE, Mini-Mental State Examination; NMSS, Non-Motor Symptoms Scale; VHI, Voice Handicap Index; GFQ, Gait and Falls Questionnaire; PDQ-8, 8-item Parkinson's Disease Questionnaire; LEDD, levodopa equivalent daily dose; GPi, globus pallidus interna; STN, subthalamic nucleus. ^*^p-value < 0.05.

Regarding the voice deterioration, an insignificant increase in the VHI score was noted after both initial GPi DBS (16.3 ± 14.7, *p* = 0.312) and rescue STN DBS (19.3 ± 11.8, *p* = 0.885) compared with the baseline (8.0 ± 16.0). In terms of the global cognitive function, the MoCA score remained comparable between initial GPi DBS and rescue STN DBS (post-GPi: 22.7 ± 2.3 vs. post-STN: 25.3 ± 2.5, *p* = 0.885) (Table 2).

### Adverse effects

No severe or persistent adverse events were documented throughout the study. Several temporary, reversible stimulation-related corticobulbar or corticospinal side effects during programming were observed, including muscle contractions, dysarthria, and eye flashes, which could be resolved by parameter adjustments.

Of note, one patient developed voice impairment 1 year after rescue STN DBS. Another patient with a 14-year disease course showed an improvement in gait after rescue STN DBS in the short term, but deterioration developed at the 2-year follow-up. Both patients achieved limited improvement after stimulation parameter adjustments.

## Discussion

In this case series, the rescue STN DBS improved off-medication motor and non-motor symptoms, as well as the health-related quality of life in PD patients with failure of the initial GPi DBS. Although the on-medication overall motor performance remained comparable between the initial GPi DBS and the rescue STN DBS, the latter substantially reduced the anti-parkinsonian medication. The clinically relevant improvement provided by rescue STN DBS was associated with an insignificant decline in speech performance, which is consistent with the current literature (Phokaewvarangkul et al., 2019; Lin et al., 2021).

Exploring the targeting of another brain structure was considered as one rescue option following the failure of the initial DBS surgery in the PD treatment. In the current literature, most of them were reported as case or case series and are summarized in Supplementary Tables 5, 6. Interestingly, the preference in choosing the type of the rescue procedure for GPi and STN is different. Most of the reported rescue procedures for poor efficacy of the initial GPi DBS require a change of GPi to STN (four studies, *n* = 15) (Allert et al., 1996; Houeto et al., 2000; Volkmann et al., 2004; Brinke et al., 2018). Only one study reported the addition of STN to GPi, but at least one GPi lead was also removed in three of four patients (Volkmann et al., 2004). In contrast, the majority of the studies reporting rescue procedures for STN DBS involved adding GPi to STN (4 studies, *n* = 7) (Allert et al., 2012; Minafra et al., 2014; Cook, 2015; Matias et al., 2016), while one study applied a substitution approach (*n* = 7) (Zhang et al., 2019).

Furthermore, another discrepancy between failures of initial GPi and STN DBS was the length of the interval between the initial surgery and failure occurrence. Consistent with this study and our previous work (Zhang et al., 2019), the current literature suggested that a worsening of motor symptoms in the short term (usually within 2–3 years) was the main reason for seeking a rescue procedure after the initial GPi DBS (Houeto et al., 2000; Volkmann et al., 2004; Brinke et al., 2018), suggesting a lack of primary efficacy of GPi DBS for these cases so that a target substitution might be more rational to offer off-medication improvements. On the contrary, a gradual waning of benefits in the long term (usually within 6–12 years) (Allert et al., 2012; Minafra et al., 2014; Matias et al., 2016; Zhang et al., 2019), appearance of refractory axial disabilities, (Minafra et al., 2014; Zhang et al., 2019) and dyskinesias (Allert et al., 2012; Minafra et al., 2014; Cook, 2015; Matias et al., 2016; Zhang et al., 2019) were primary reasons for concerning a rescue procedure after the initial STN DBS, suggesting a conclusive efficacy of STN DBS followed by long-term stimulating- and/or disease progression-related disabilities, to which GPi DBS may provide additional benefits in the on-medication condition (Munhoz et al., 2014). Only one study had switched STN to GPi following a gradual waning of the efficacy of STN DBS with a mean reoperation interval of 6.3 years (Zhang et al., 2019). This decision might be partially due to the economic issue that an additional out-of-pocket stimulator should be implanted, which might be unaffordable for patients.

Another intriguing finding is that rescue DBS procedures were primarily conducted in patients with early-onset PD, in both our study and previous research. In our study, these six patients had an average onset age of 10 years younger than other patients who underwent GPi DBS (42.7 ± 9.8 vs. 53.3 ± 8.5). In previous studies involving rescue DBS (both GPi to STN and vice versa), the average onset age was 40.1 years (our study included). This observation preliminarily suggested that early disease onset and longer disease duration may be potential risk factors contributing to initial target failure and the need for target revision. Additionally, early-onset PD patients were previously associated with specific genetic mutations, but the genetic test was not included in current research. Therefore, the relationship between gene mutations and the efficacy of stimulation targets is unknown but worthy of future investigation.

In the clinical practice, the following aspects should be considered in case of lack of initial efficacy of DBS: (1) an inadequate patient screening, e.g., a poor levodopa responsiveness on non-tremor parkinsonian motor symptoms; (2) an off-target lead implantation; and (3) suboptimal postoperative medication and parameter adjustments. However, not all studies in the current literature have reported the screening criteria or the coordinates of active contacts (Supplementary Table 5). In this cohort, all patients were levodopa-responsive preoperatively, all GPi lead placements were verified to exclude the apparent electrode displacement, and efforts were made to optimally adjust parameters and medications prior to the rescue surgery. Four out of six patients were classified as responders after the rescue STN DBS compared to the initial GPi DBS, suggesting that the rescue STN DBS could provide additional benefits despite an on-target placement of initial GPi leads. However, inconsistent with this work, Brinke et al. (2018) concluded that the objective efficacy of the rescue STN surgery might be limited if the GPi leads were correctly placed during the initial surgery, but lead positions were not demonstrated in the article. This discrepancy might be further elucidated in future studies with more advanced neuroimaging and targeting techniques. Indeed, Volkmann et al. have postulated that the lack of initial efficacy of GPi DBS may be related to a larger size and the functional segregation of this nucleus, which was only partially and insufficiently modulated in these cases (Volkmann et al., 2004). On the contrary, for patients experiencing short-term effectiveness but long-term reduction in therapeutic effects following STN DBS, it may be attributed to adaptive changes induced by prolonged stimulation. Modulating another over-expressed pathway (GPi) could potentially introduce additional benefits (Minafra et al., 2014).

Even though the long-term efficacy of DBS has been confirmed by numerous studies (Castrioto et al., 2011; Li et al., 2013; Zhou, 2019; Cavallieri et al., 2021), some research has reported negative outcomes, particularly concerning axial symptoms (Castrioto et al., 2011). The challenge remains to sustain long-term therapeutic effects of DBS. In this context, therapeutic interventions targeting plasticity-related mechanisms (Shen et al., 2003; van Hartevelt et al., 2014; Awad, 2021) may yield beneficial effects. Some novel temporal stimulation patterns were computationally designed to counteract the abnormal synchronization of neuronal activity (Krauss et al., 2021). For instance, coordinated reset (CR) stimulation (Adamchic et al., 2014), which delivers brief high-frequency pulse trains through different stimulation contacts, exploited spike timing-dependent plasticity (STDP)-induced biostability to shift the dynamics of pathological networks toward physiological attractor states (Madadi Asl et al., 2022a,b, 2023). The long-lasting effects of CR stimulation on motor symptoms in PD were verified clinically (Adamchic et al., 2014; Syrkin-Nikolau et al., 2018), indicating the potential of temporal patterns to achieve an enhanced efficiency of stimulation and prolong symptom relief through the control of plasticity (Krauss et al., 2021).

DBS targets should be tailored primarily to the specific symptom characteristics and treatment goals. From the contrarian perspective of poor initial efficacy in a small number of patients, studies on rescue therapies corroborated the subtle difference between STN and GPi and provided evidence for individualized target selection with prolonged observation. However, the clinical characteristics of these patients were difficult to clearly summarize the small sample reported so far. From the insufficient evidence available, a substitution to the STN target may be considered when the initial GPi DBS is not effective in the short term after parameter refinements. In contrast, additional GPi stimulation can be considered in cases of axial deterioration or uncontrolled dyskinesias with long-term STN stimulation. This inference warranted further validation in multicentric cohorts with larger samples and long-term follow-ups. In addition, some authors have also investigated the feasibility and efficacy of initial bilateral, dual-target DBS on GPi and STN (Peppe et al., 2004; Mazzone et al., 2005; Chang et al., 2021; Mitchell et al., 2022). However, these studies were designed primarily for exploratory scientific purposes and were limited by small sample size and potential increased surgical risks and economic burden. Therefore, from our perspective, the initial GPi-STN dual-target DBS would be unnecessary.

This study has several limitations. Firstly, not all patients who underwent GPi DBS at our center were closely followed up with complete and detailed clinical data, making it challenging to compare these “rescued” patients to the entire cohort. Secondly, the stimulation effect for off-stimulation evaluations washed out for 1 h, which was clinically feasible but might be insufficient for axial symptoms. Thirdly, the motor fluctuation and dyskinesia were not quantitatively evaluated, which could potentially under-evaluate the benefits of the initial GPi DBS. However, despite the absence of dyskinesia and motor fluctuations following GPi DBS, the unresolved primary complaints, such as rigidity and bradykinesia, were remained, which contributed to our characterization of failures in GPi DBS.

## Data availability statement

The raw data supporting the conclusions of this article will be made available by the authors, without undue reservation.

## Ethics statement

The studies involving humans were approved by Ethics Committee of the Ruijin Hospital Shanghai Jiao Tong University School of Medicine. The studies were conducted in accordance with the local legislation and institutional requirements. The participants provided their written informed consent to participate in this study. Written informed consent was obtained from the individual(s) for the publication of any potentially identifiable images or data included in this article.

## Author contributions

ZZ: Conceptualization, Investigation, Writing—original draft. PH: Conceptualization, Data curation, Writing—review & editing. ZL: Investigation, Methodology, Writing—review & editing. YP: Investigation, Project administration, Writing—review & editing. XW: Software, Validation, Writing—review & editing. CZ: Investigation, Resources, Supervision, Writing—review & editing. BS: Project administration, Supervision, Validation, Writing—review & editing. DL: Conceptualization, Funding acquisition, Project administration, Supervision, Writing—review & editing.
