# Supplementary material for: Rescue subthalamic stimulation after unsatisfactory outcome of pallidal stimulation in Parkinson's disease: a case series and review
Source: Front Aging Neurosci. 2024 Jan 9;15:1323541. doi: 10.3389/fnagi.2023.1323541 (PMC10803461; doi:10.3389/fnagi.2023.1323541)
Supplement: Supplementary file 1 [file Data_Sheet_1.PDF]

Supplementary material

Supplementary Figure 1. DBS lead reconstruction in the Montreal Neurological Institute (MNI) space.

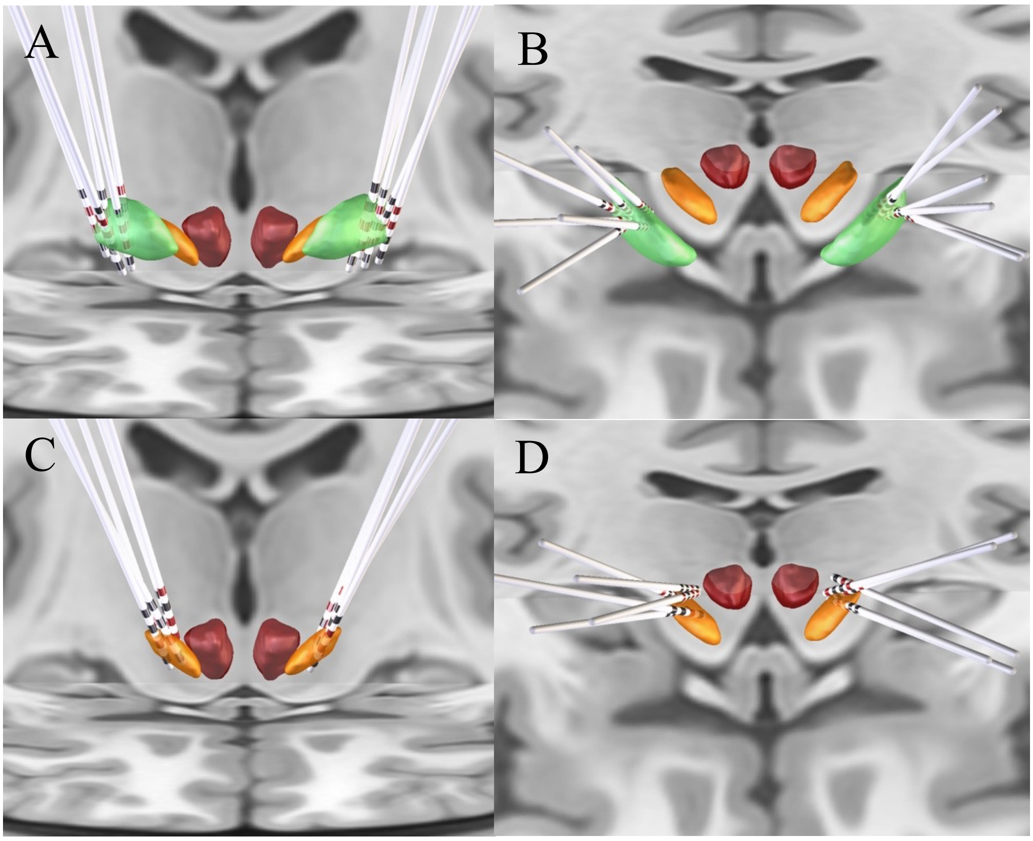

Active contacts were marked in red. the yellow, red, and green masses delineate the STN, red nucleus, and GPi, respectively.

Supplementary Table 1. Rationales for two times of DBS surgery (N = 6).

	Main symptoms of complaints (N = 6)	
**Patients**	**Before initial GPi DBS**	**After initial GPi DBS /Before rescue STN DBS**
1	Bradykinesia, rigidity, levodopa induced dyskinesia	Bradykinesia, rigidity, shorten "on" period
2	Bradykinesia, rigidity, levodopa induced dyskinesia, muffled speech	Bradykinesia, rigidity, muffled speech
3	Bradykinesia, rigidity, levodopa induced dyskinesia, gait impairment	Bradykinesia, shorten "on" period
4	Bradykinesia, rigidity, gait impairment	Bradykinesia, rigidity, painful dystonia in "off" period
5	Bradykinesia, rigidity, freezing of gait	Bradykinesia, rigidity, painful dystonia in "off" period, gait impairment, symptom-related depression and anxiety
6	Bradykinesia, rigidity, levodopa induced dyskinesia	Bradykinesia, rigidity, shorten "on" period

Abbreviations: GPi, globus pallidus interna; STN, subthalamic nucleus; DBS, deep brain stimulation.

Supplementary Table 2. Stimulation parameters of GPi DBS and STN DBS at the last follow-up.

Patient*	GPi-DBS				STN-DBS			
	Contact	PW (μs)	Freq (Hz)	Amp (V)	Contact	PW (μs)	Freq (Hz)	Amp (V)
1 Right*	C+1-2-	60	125	3.45	C+1-	60	130	2.8
1 Left#	C+9-	60	125	3.5	C+8-9-	60	130	2.9
2 Right	C+1-2-	60	160	3.75	C+1-/C+3-	60	105	2.35/2.5
2 Left	C+8-9-	60	160	2.5	C+9-/ C+11-	60	105	3.15/2.5
3 Right	C+1-2-	60	145	3.35	C+0-/C+1-	60	115	2.7/3.0
3 Left	C+9-11-	60	145	3.45	11+10-9-	60	115	3.3
4 Right	C+1-3-	60	145	2.75	C+1-	60	115	3.2
4 Left	C+10-11-	60	145	2.15	C+8-9-/C+11-	60	115	2.75/1.5
5 Right	C+2-3-	60	160	2.75	C+1-	60	160	1.75
5 Left	C+10-11-	90	160	3.65	C+9-10-	60	160	2.0
6 Right	C+1-2-	60	145	3.15	C+0-1-/ C+3-	60/ 70	105	2.8/ 2.5
6 Left	C+8-9-	60	145	3.25	C+9-/ C+11-	60/ 70	105	2.3/ 2.0

* Right means the parameters in right hemisphere. Left means the parameters in left hemisphere.

Abbreviations: GPi, globus pallidus interna; STN, subthalamic nucleus; DBS, deep brain stimulation.

Supplementary Table 3. Off-medication MDS UPDRS-III total score and sub-scores at different evaluation visits.

MDS UPDRS-III	Baseline	GPi DBS	ΔGPi-baseline	*P*-value	STN DBS	ΔSTN-baseline	*P*-value	ΔSTN-GPi	*P*-value
**Total score**	58.2 ± 17.7	51.3 ± 4.5	-11.7%	0.361	36.3 ± 8.3	-37.5%	0.003*	-29.2%	0.006*
**Rigidity**	12.8 ± 5.3	11.5 ± 4.0	-10.4%	0.658	6.7 ± 2.5	-48.1%	0.081	-42.0%	0.013*
**Bradykinesia**	27.0 ± 11.3	25.5 ± 6.1	-5.6%	0.691	18.5 ± 9.2	-31.5%	0.006*	-27.5%	0.043*
**Tremor**	5.8 ± 7.6	4.2 ± 4.7	-28.6%	1.000	3.5 ± 2.6	-40.0%	0.936	-16.0%	0.706
**Speech**	1.7 ± 0.5	0.8 ± 0.8	-50.0%	0.069	1.0 ± 0.9	-40.0%	0.190	20.0%	0.611
**Axial sign**	10.8 ± 5.0	9.3 ± 2.5	-13.8%	0.357	6.7 ± 1.0	-38.5%	0.088	-24.6%	0.082

Abbreviations: MDS UPDRS-III, Movement Disorder Society-Unified Parkinson Disease Rating Scale part III; GPi, globus pallidus interna; STN, subthalamic nucleus.

Supplementary Table 4. On-medication MDS UPDRS-III total score and sub-scores at different evaluation visits.

MDS UPDRS-III	Baseline	GPi DBS	ΔGPi-baseline	*P*-value	STN DBS	ΔSTN-baseline	*P*-value	ΔSTN-GPi	*P*-value
**Total score**	22.7 ± 6.2	29.2 ± 13.0	30.1%	0.178	28.3 ± 10.0	25.0%	0.109	-4.0%	0.705
**Rigidity**	4.7 ± 3.4	7.0 ± 3.9	50.0%	0.225	5.7 ± 2.1	21.4%	0.403	-19.0%	0.414
**Bradykinesia**	12.2 ± 5.6	15.5 ± 7.8	27.4%	0.348	14.3 ± 8.5	17.8%	0.546	-7.5%	0.523
**Tremor**	1.7 ± 2.3	1.3 ± 2.0	-20.0%	0.737	2.0 ± 1.9	20.0%	0.805	50.0%	0.617
**Speech**	0.7 ± 0.5	1.0 ± 0.6	50.0%	0.386	1.3 ± 0.8	100%	0.140	33.3%	0.363
**Axial sign**	3.5± 2.2	4.7 ± 2.7	33.3%	0.302	5.0 ± 1.4	42.9%	0.060	7.1%	0.732

Abbreviations: MDS UPDRS-III, Movement Disorder Society-Unified Parkinson Disease Rating Scale part III; GPi, globus pallidus interna; STN, subthalamic nucleus.

Supplementary Table 5. Demographic and clinical characteristics of studies reviewed.

Study	Sample size (/total No. of patients receiving surgeries if reported)	Intervention (bilaterally if not specified)	Age at initial surgery (year)	Disease duration at initial surgery (year)	Time between two operations (year)	Effective time for initial surgery	Last follow-up after rescue surgery	Electrode type
**Initial GPi DBS followed by rescue STN DBS**								
Houeto et al. (2000)	2 (/5)	GPi → STN	64, 46	18, 12	2.25, 3.25	3 months	6 months	GPi: 3387; STN: 3389
Volkmann et al. (2004)	4 (/11)	GPi + STN[#]	45.8 ± 8.6[&]	11.2 ± 2.7[&]	2–3 years	1.5 years	2 years	GPi: 3387; STN: NA
Allert et al. (2010)	1 (/9)	GPi → STN	38	8	10	3–5 years	1 year	NA
ten Brinke et al. (2018)	8 (/65)	GPi → STN	51.6 ± 8.9	9.5 ± 3.9	2.25 ± 1.5	several months: n=5<br>1 year: n=2<br>4.5 years: n=1	mean 16.4 months	NA
**Initial STN DBS followed by rescue GPi DBS**								
Allert et al. (2012)	1	STN + GPi	50	20	8	2 years	6 months	NA
Minafra et al. (2013)	3	STN + GPi[$]	54, 59, 50	10, 15, 7	8	7 years	2 years	NA
Cook et al. (2015)	2	STN + GPi	50, 27	20, 1	1, 6	perioperative: n=1<br>2 years: n=1	NA	STN: 3387; GPi: 3387
Matias et al. (2016)	1	STN + GPi	41	8	12	4 years	6 months	NA
Zhang et al. (2019)	7 (/180)	STN → GPi	60.1 ± 7.6	16.0 ± 2.2	mean 6.3	NA	6 months	STN: NA; GPi: 3387

[#]with removal of at least one GPi lead in 3 patients；[&]for total cohort (n=11)；[$]STN Leads were kept but stimulation was turned off.

Abbreviations: GPi, globus pallidus interna; STN, subthalamic nucleus; DBS, deep brain stimulation.

Supplementary Table 6. Preoperative complaints and surgical outcomes of studies reviewed.

Study	Main complaints before initial surgery	Main reasons for rescue surgery	Main outcomes of rescue surgery
**Initial GPi DBS followed by rescue STN DBS**			
Houeto et al. (2000)	severe motor fluctuations and levodopa-induced dyskinesias	main initial complaints unresolved	1) improvements in UPDRS-III score (off-medication: 53%, 79%), fluctuations (UPDRS-IV: 50%, 100%), and dyskinesias (UPDRS-IV: 78%, 78%)<br>2) reduction of LEDD (89%, 100%)
Volkmann et al. (2004)	NA	a decline of efficacy of initial GPi DBS, device-related complications	1) improvement in UPDRS-III score (off-medication: 32%) and dyskinesias<br>2) reduction of LEDD (70%)
Allert et al. (2010)	severe motor fluctuations and levodopa-induced dyskinesias	considerable psychiatric, non-motor fluctuations, dystonic dyskinesias	1) improvement in UPDRS-III score (off-medication: 64%), UPDRS-II score (70%), fluctuations, dyskinesias, and psychotic symptoms<br>2) reduction of LEDD (100%)<br>3) reduction of energy consumption of the stimulator
ten Brinke et al. (2018)	severe motor fluctuations, axial disabilities, and dyskinesias	an initial lack of efficacy (mostly) despite a satisfactory placement of GPi leads	1) only two of the eight patients showed objective clinical off-medication UPDRS-III score improvement > 30%<br>2) reduction of UPDRS-III (off-medication: mean 21%), with two postoperative scores missing

			3) removal of DBS system in one patient due to infection
**Initial STN DBS followed by rescue GPi DBS**			
Allert et al. (2012)	severe motor fluctuations and peak-dose dyskinesias	a decline of efficacy of initial STN DBS	1) improvements in akinesia, motor fluctuations, and dyskinesias
Minafra et al. (2013)	motor fluctuations, levodopa-induced dyskinesias, dopaminergic dysregulation syndromes	axial disabilities, motor fluctuations	1) no significant change in off-medication UPDRS-III score (mean -12%)<br>2) improvements in dystonia, dyskinesias and the subsequent axial stabilities.<br>3) no significant change in LEDD (mean -7%)
Cook et al. (2015)	tremor-dominant PD with motor fluctuations and dyskinesias, young-onset PD	disabling and painful dystonic dyskinesias	1) suppression of dyskinesias<br>2) dual STN and GPi stimulation is required
Matias et al. (2016)	motor fluctuations, axial disabilities, and dyskinesias	dystonic symptoms involving the trunk and both extremities	1) improvements in MDS UPDRS-III (on-medication: 60%), MDS UPDRS-II (44%), NRS-11(42%), and EQ-5D scores (82%)<br>2) reduction of LEDD (55%)
Zhang et al. (2019)	NA	severe axial disabilities, motor fluctuations and dyskinesia	1) improvements in UPDRS-III (on-medication: 44%), UPDRS-IV (52%), VHI (51%), and PDQ-39 scores (34%)<br>2) reduction of a LEDD (34%)

Abbreviations: GPi, globus pallidus interna; STN, subthalamic nucleus; DBS, deep brain stimulation; MDS UPDRS-II, -III, -IV, Movement Disorder Society-Unified Parkinson Disease Rating Scale part II, III, IV; UPDRS-II, -III, -IV, Unified Parkinson Disease Rating Scale part II, III, IV; LEDD, levodopa equivalent daily dose; NRS-11, Numeric Rating Scale-11; EQ-5D EuroQol 5 Dimensions Questionnaire; PDQ-39, 39-item Parkinson's Disease Questionnaire; VHI, Voice Handicap Index.
